# Hydronaphthol—With Suggestions for Its Use in the Treatment of the Dental Pulp, Alive or Dead

**Published:** 1898-06

**Authors:** Sidney S. Stowell

**Affiliations:** Pittsfield, Mass.


ARTICLE VI.

### HYDRONAPHTHOL—WITH SUGGESTIONS FOR ITS USE IN THE TREATMENT OF THE DENTAL PULP, ALIVE OR DEAD.

SIDNEY S. STOWELL, D. D. S., PITTSFIELD, MASS.

**Read before the First District Dental Society of the State of New York, March 9, 1897.**

That I have discovered the elixir of life I do not claim, for it is appointed unto all men, and some teeth, once to die. But there is a balm in Gilead, and it is that which I have brought you; its name is "hydronaphthol." The makers state as follows : "Hydronaphthol is a perfectly harmless, odorless, non-poisonous, non-corrosive, antiseptic, germicide, perservative, anti-forment and disinfectant. Its name indicates a descriptive fancy word which is used to designate a certain secondary compound derived from beta-naphthol by reaction which changes its molecular constitution by means of inorganic compounds that do not enter the molecule, and are finally removed, whereby ninety per cent. of a product is obtained, melting at 116° F."

Hydronaphthol is soluble in the following proportions: In alcohol, about 1 to 2 parts; in hot water, about 1 to 300 parts; in cold water, about 1 to 1,100 parts; in olive oil, about 1 to 20 parts; in ether, about 1 to 2 parts; in chloroform, about 1 to 2 parts. It is also freely soluble in benzol and the fixed oils. Alkaline solutions dissolve it readily, but greatly reduce its antiseptic properties.

Hydronaphthol possesses one-fifth the antiseptic strength of mercury-bichlorid, double the strength of beta-naphthol and iodoform, three times the strength of salicylic acid, and about fourteen times the strength of carbolic acid. Unlike all these antiseptics it is absolutely non-poisonous, and can be used with perfect impunity as a preservative in cases where no other antiseptic could be used at all. It is claimed that a solution of one to ten thousand will prevent fermentation, but, to arrest of putrefactive germs already formed, stronger solutions are necessary.

Whatever hydronaphthol may be, scientific chemical experts have shown that *it is not* beta-naphthol, and medical experts are satisfied that it is a product which produces a physiological action in which all the disadvantages arising from the use of other hydro-compounds of naphthalin are entirely removed. Thorough and exhaustive tests and experiments by painstaking, scientific investigators in hospital and private practice have established its claim to a prominent and permanent place in the field of antiseptics, because in surgical dressings it is sufficiently soluble to effect thorough antisepsis of the wound discharge, and sufficiently insoluble not to be readily washed away from the dressing by the discharge. It is non-toxic, and may be given in three-grain doses as an intestinal antiseptic in cases of typhoid fever and cholera. Leading American and English physicians have published statements to this effect. In germicidal powers it ranks second to mercury-bichlorid, but being non-poisonous, non-corrosive and harmless, it is preferable to that substance not only in surgical practice, but in the arts also as a preventive of fermentation, etc. It preserves anatomical and pathological specimens in their natural condition, preventing shrinkage or decay.

A. B. Griffiths, Ph. D., F. R. S. E., in his "Researches on Micro-organisms," writes: "Hydronaphthol is of great value as an antiseptic agent in surgical operations, etc. A solution one to eleven hundred destroys bacillus subtilis, micrococcus tetragenus, micrococcus prodigiosus, bacillus anthracis and its spores, sarcina lutea, and other microbes, for they cannot be cultivated after its action."

Dr. J. C. McCoy, New York, writes: "After a daily use of hydronaphthol as an antiseptic and disinfectant for several years, I consider it pre-eminent as a dental antiseptic and disinfectant, and for practical purposes it will supersede all other antiseptics in the practice of dental surgery."

Dr. C. A. Meeker, Newark, N. J., writes: "I am greatly pleased with the antiseptic properties of hydronaphthol. I believe them to be far higher than carbolic acid and other antiseptics, and the fact of its being non-poisonous is a great factor in favor of its use in general dental practice. I have used it for several years and shall so continue."

Alex. Edington, M. B., C. M., Edinburgh, writes at length of his experiments with hydronaphthol in hospital surgery and private practice, comparing its efficiency with carbolic acid, claiming great superiority for hydronaphthol and concludes by saying, "there can be no doubt of the fact that in this agent we have an ideal antiseptic, and a solution of one in three hundred in warm water possesses very great germicidal power, and such will be found of the greatest service in washing out septic cavities and wounds."

Dr. John I. Hart, New York, writes to me: "I have used hydronaphthol, saturated solution in alcohol, in root-canals; also mixed in equal parts with zinc oxid, then with zinc chlorid as a permanent root filler; also mixed with xinc oxid and creosote or oil of cloves, placing the paste thus formed over a layer of dentin too close to the pulp to remove, and over this paste flowing oxyphosphate cement, also with tannin and glycerol in pyorrhea-pockets."

That I might prove it well I have used the drug constantly for more than five years to the exclusion of nearly every other antiseptic agent, and with such phenomenal success that I deem it my duty to the profession which I represent, and to humanity at large, that I present its merits here. I use hydronaphthol principally in two forms, the twenty-five per cent. alcoholic solution and the powdered form. In all cases where an antiseptic solution is required in dentistry I use the alcoholic solution, often reducing it with glycerol or water as the case may require. In the treatment of a putrescent pulp-canal, the penetrating property of the alcohol and its affinity for moisture carries it laden with its hydronaphthol in solution to the remotest nook and corner of the pulp-chamber and canals however small, even to the apex and through into the soft tissues, as well as into the tubuli of the tooth. The alcohol having thus penetrated every part it at once evaporates, leaving the hydronaphthol to do its perfect work of disinfection.

We all know with what fear aud dread we have opened into a pulpless tooth of long standing, having no open fistula, and what dire results have followed, supposed to be the result of a multitude of hungry microbes rushing into a fertile field. An inflammation is at once set up which is almost unbearable, the quickening pulse and rapid breathing soon indicate a high fever, and often for two or three days or a week, the patient, whether a robust man or a delicate woman, will be confined to the bed in a dark room, there to writhe in agony until suppuration of the inflamed part takes place and the pus burrows its painful path through the plate of bone and soft tissues, and finally breaks and discharges. In the meantime the physician and dentist have been in almost constant attendance, using all the known remedies for such cases, and neither doctors nor medicines have really done any good, other than to encourage and speak good cheer to the suffering martyr that for all this agony he will still retain the tooth, that it may yet become useful. We have all been through this experience, either personally or with our patients, and all have realized how powerless we were with all our drugs to relieve our patient's suffering. Gentlemen, I make the statement here that when an old pulpless tooth is opened into, if the alcoholic solution of hydronaphthol is at once placed in the pulp-canal, the conditions referred to are *absolutely impossible*.

I will now refer to another use of hydronaphthol which I have found of priceless value, and so far as my research has extended I may claim originality. I refer to the combination of the powdered hydronaphthol with the zinc oxid of our common oxyphosphate cement. I combine in parts one-quarter of hydronaphthol to three-quarters zinc oxid, mixing this combination with the phosphoric acid, and using as any oxyphosphate cement for pulp-capping and for protecting permanently all exposed, sensitive or soft parts in live teeth as an antiseptic, non-irritating lining for all classes of fillings. I first bathe the cavity with the alcoholic solution, which penetrates the soft leathery decay as well as the tooth-structure; the alcohol soon evaporates, leaving a deposit of hydronaphthol in the cavity. I now either line the cavity or fill it entirely with the hydronaphthol cement, which is rendered non-irritant and antiseptic by the presence of the hydronaphthol incorporated into it; I sometimes incorporate a little oil of cloves or carbolic acid with this cement, which acts favorably as a local anesthetic; with this addition the foregoing treatment may be applied to a tooth with a pulp slightly or nearly exposed, which has ached some or may be aching at the time of presentment, with almost positive assurance of successful results and without pain to the patient, who should be instructed to call again in a few months for a more permanent filling, when the tooth will be found to be quite free from sensitiveness; this cavity may now be more thoroughly prepared and a more permanent filling placed in it, lining the cavity again with the combination cement. If, however, the cement is not badly worn, it may be left for a much longer time with still better results.

The addition of hydronaphthol to the cement in the proportion mentioned reduces its wearing quality but slightly if it is mixed hard, but as we use the cement quite soft in the treatment of such cases, its wearing quality is of course impaired. Now whatever of moisture may remain in the soft decay or in the tooth structure, or may be secreted by the pulp (there being no such thing as a permanently dry tooth in the mouth), on coming in contact with and acting upon the cement-lining, immediately sets free more hydronaphthol which at once acts to keep the parts in a thoroughly antiseptic condition, while good old Dame Nature, ever ready and willing to heal and restore her diseased and lost parts when given an opportunity, goes quietly and peacefully on with her perfect work of recalcification of the affected tooth.

I will say that the results thus obtained have been little short of marvelous, as I have taken liberties with hypersensitive and soft teeth by leaving the soft sensitive decay undisturbed in the cavity, treating it as before mentioned to the delight and comfort of my patient, and assurance to myself that I have put nature at her best to restore the affected part to a healthy and normal condition, less the part lost by the first attack of the deadly microbe. I make the claim, and in good faith, that by this treatment in general practice I will save more teeth with living healthy pulps and withvastly greater comfort to my patient than any one else can do by thorough excavating, as suggested by Dr. Ottolengui in his recent discussion of Dr. Williams' paper. I make still further use of hydronaphthol by combining it with the zinc oxychlorid, in the same proportion as with the oxyphosphate cement, as a permanent root-canal and pulp-chamber filler; especially is it valuable in this form where we are not sure that we have thoroughly cleansed the canals, as is very often the case with molar teeth when a "mummifying" agent is required. *Dental Cosmos.*

